# Expression and phase separation potential of heterochromatin proteins during early mouse development

**DOI:** 10.15252/embr.201947952

**Published:** 2019-11-07

**Authors:** Manuel Guthmann, Adam Burton, Maria‐Elena Torres‐Padilla

**Affiliations:** ^1^ Institute of Epigenetics and Stem Cells (IES) Helmholtz Zentrum München München Germany; ^2^ Faculty of Biology Ludwig‐Maximilians Universität München Germany

**Keywords:** development, epigenetics, heterochromatin establishment, phase separation, Chromatin, Epigenetics, Genomics & Functional Genomics, Development & Differentiation

## Abstract

In most eukaryotes, constitutive heterochromatin is associated with H3K9me3 and HP1α. The latter has been shown to play a role in heterochromatin formation through liquid–liquid phase separation. However, many other proteins are known to regulate and/or interact with constitutive heterochromatic regions in several species. We postulate that some of these heterochromatic proteins may play a role in the regulation of heterochromatin formation by liquid–liquid phase separation. Indeed, an analysis of the constitutive heterochromatin proteome shows that proteins associated with constitutive heterochromatin are significantly more disordered than a random set or a full nucleome set of proteins. Interestingly, their expression begins low and increases during preimplantation development. These observations suggest that the preimplantation embryo is a useful model to address the potential role for phase separation in heterochromatin formation, anticipating exciting research in the years to come.

## Graphical abstract

Proteins of the constitutive heterochromatin proteome tend to contain intrinsically disordered regions. Their expression correlates with chromocenter formation and reduced cellular plasticity during mouse pre‐implantation development.

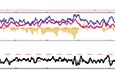

## Introduction

In eukaryotes, around 145 basepairs of DNA are wrapped around octamers of the four canonical histones H2A, H2B, H3 and H4 to form the nucleosome. The nucleosome is the building block of the chromatin, which in addition includes other chromatin‐associated proteins that bind nucleosomes and also the linker histone H1. Functionally, chromatin has been traditionally divided into two categories: hetero‐ and euchromatin 1, which were first recognised cytologically by Emil Heitz 2. Heterochromatin appeared as regions of the nucleus that do not decondense after mitosis, which he considered to be a non‐functional part of the genome. Nowadays, the definition of heterochromatin has broadened to include features such as (i) histone modifications such as histone 3 lysine 9 trimethylation (H3K9me3), H3K27me3, DNA methylation and potentially also H3K56me3 3, 4; (ii) a (mostly) transcriptionally silent state; (iii) a late replicating nature; (iv) an electron‐dense and condensed state in electron microscopy 5, and more recently (v) a higher resistance to sonication 6. Heterochromatin can be further broadly divided into constitutive heterochromatin—which is located at centromeric and telomeric regions, as well as at most repeat elements throughout most eukaryotic genomes—and facultative heterochromatin, which harbours the H3K27me3 mark and often localises to temporally or spatially regulated genes 5.

Over the last two decades, a rather unified model for constitutive heterochromatin establishment has emerged whereby the Suv39h1/h2 (Su(var)9‐1) enzymes initiate a feedback cascade by catalysing H3K9me3, which in turns recruits heterochromatin protein 1 (HP1) proteins, primarily through their chromodomain 7, 8, 9. Downstream recruitment of Suv420h1/h2 (Su(var)4‐20) reinforces a heterochromatic loop by catalysing H4K20me3 10, while as yet unknown enzymes deposit H3K64me3 11. Subsequent recruitment of Suv39h1/h2 by both HP1 and H3K9me3 enables spreading and amplification of the heterochromatin domain. In addition, RNA‐mediated interactions of HP1 and the Su(var) enzymes themselves have also been implicated in maintaining constitutive heterochromatin in mouse, human and yeast 12, 13, 14, 15. However, relatively little is known about the mechanisms that direct heterochromatin formation *in vivo*, at the beginning of development.

It has recently been suggested that heterochromatin can form by phase separation through the local accumulation of HP1α 16, 17. Phase‐separated compartments appear as immiscible liquid droplets that emerge through multivalent, weak interactions between biological polymers, which can be either proteins or nucleic acids 18, 19. Multivalent interactions can be provided by intrinsically disordered domains (IDRs) or structured domains. Liquid droplets can undergo fission, coalesce into larger droplets and relax to their original spherical shape after shear stress 20, 21. Since the discovery that P granules form by liquid–liquid phase separation in the *Caenorhabditis elegans* germline around 10 years ago, many studies have shown that several membrane‐less organelles may in fact form through phase separation 22, 23, 24, 25, 26. These include the nucleolus, which has physical properties of a phase‐separated liquid‐like droplet formed of several immiscible liquid sub‐compartments 21, 27, but also stress granules and paraspeckles 28, 29 as well as cajal bodies 23. More recently, some studies have also suggested a role for phase separation in transcription initiation, by facilitating the recruitment of the transcriptional machinery 30, 31, 32, 33, 34, 35. Similarly, liquid–liquid phase separation was suggested to play a role in facultative heterochromatin formation by enabling the assembly of the polycomb repressive complex 1 36.

In the phase‐separation‐based model for constitutive heterochromatin formation 16, 17, 37, the binding of HP1α to H3K9me3 would lead to a local increase in HP1α concentration, which in turn would nucleate a phase‐separated compartment that could then grow and fuse, enabling the formation of constitutive heterochromatin. The liquid–liquid phase separation biophysical properties would also explain the selective exclusion of certain proteins from these heterochromatin compartments. In such a model, exclusion from domains may be due to the inability to interact with phase‐inclusive components, but it can also result from the emergent biophysical properties of the domain. However, a recent report shows that IDR‐rich liquid condensates tend to exclude chromatin, which is at odds with the proposed growth and fusion of phase‐separated heterochromatin compartments. In fact, when promoting droplet formation at heterochromatin using a synthetic “CasDrop” approach, condensates appear at the periphery of such regions 38. Thus, these conceptual frameworks to understand the formation and physical properties of heterochromatic genomic regions are still in their early days, and have not yet incorporated all the additional proteins known to be present at constitutive heterochromatin, and which may therefore play a role in regulating heterochromatin establishment.

How and whether these mechanisms operate in the early mammalian embryo at the onset of epigenetic reprogramming are unknown. Even though heterochromatin has been extensively studied, little is known about its biophysical properties as well as the mechanisms that underlie heterochromatin formation, as opposed to maintenance, *in vivo*. Here, we have undertaken an analysis to investigate the properties of heterochromatin‐associated proteins and their potential to phase separate as well as their expression pattern at the earliest developmental stages in the mouse embryo. Finally, we propose possible avenues for addressing phase separation as a potential mechanism for heterochromatin formation at the beginning of development.

## Results and Discussion

Several mass spectrometry studies have been carried out in mammalian cells to better understand the pathways involved in constitutive heterochromatin maintenance and integrity. Most of them focused on the identification of proteins that bind H3K9me3 using peptides or modified nucleosomes pulldowns 39, 40, 41 or chromatin immunoprecipitation 42, 43, 44. More recently, heterochromatin proteins have been identified by mass spectrometry of the sonication‐resistant fraction of the chromatin 6. Functionally, however, much of our knowledge on heterochromatin stems from genetic screens in model organisms including *Schizosaccharomyces pombe*,* C. elegans* and *Drosophila melanogaster* 45, 46, 47. In *Drosophila*, position‐effect variegation analyses have identified proteins important for heterochromatin maintenance and/or spreading 48. Likewise, genetic screens in *S. pombe* have uncovered genes involved in heterochromatin integrity using a pericentromeric insertion of the ade6^+^ reporter for example 49. In *C. elegans*, many repressors have been identified in screens for defects in vulva development or nuclear peripheral localisation 46, 50.

In an effort to identify the most relevant protein components of constitutive heterochromatin—and thereby potential proteins that may promote heterochromatin phase separation—we undertook a bioinformatic analysis, initially based on 7 mass spectrometry studies performed in mammalian cells 6, 39, 40, 41, 42, 43, 44. We focused primarily on H3K9me3 as a proxy for constitutive heterochromatin, since it is its most prevalent mark across most, albeit not all, eukaryotes. We selected proteins as heterochromatic based on their ability to bind H3K9me3‐modified peptides, H3K9me3‐modified nucleosomes with and without methylated DNA, or to their presence in the sonication‐resistant fraction of the chromatin. Our analysis of all these studies revealed 672 proteins identified as heterochromatic by at least one study, with 148 of these proteins being present in more than one study (Table EV1). To increase stringency in our selection, we then explored the conservation across evolution of the proteins identified by mass spectrometry. For this, we searched for the ortholog genes encoding the 672 proteins in *Danio rerio*,* S. pombe*,* D. melanogaster* and *C. elegans*. Our results show that 205 (31%) genes had orthologs in all the species that we investigated. In addition, 36 (24%) of the 148 genes coding for the proteins found in more than one mass spectrometry study had orthologs in all species (Table EV1). Among these, 36 genes are the well‐characterised *Cbx1*,* Cbx3* and *Cbx5,* which encode the three mammalian HP1 isoforms known to bind H3K9me3 and to play a role in constitutive heterochromatin maintenance and/or spreading. We thus speculate that a thorough investigation of the remaining 33 genes will lead to the discovery of other proteins that may play a role in constitutive heterochromatin.

Because a biochemical identification does not necessarily imply that these proteins and their corresponding orthologs functionally regulate heterochromatin formation and/or maintenance, we mined our results against datasets derived from previous genetic screens. This was possible in three species (*S. pombe*,* D. melanogaster* and *C. elegans*) but not in *D. rerio*, as we were unable to find publicly available compilations of screens in this species 46, 48, 49. Interestingly, we found very little overlap between the 672 proteins identified based on the biochemical studies performed with mammalian cell culture models, and the genetic screens across other model organisms. In fact, only *Cbx1*,* Cbx3* and *Cbx5* were common across all datasets and species. This raises interesting questions, as to whether non‐“core” heterochromatin proteins in different species may be important to potentially specify additional heterochromatin features. Alternatively, redundancy could potentially prevent identification of proteins in *in vivo* screens. Due to the small number of hits obtained through the analysis of genetic screenings, we decided to perform our downstream analyses below on the common 148 proteins identified from the biochemical studies, which, for simplicity, will be referred to as heterochromatic proteins hereafter.

### The physical properties of phase separation and heterochromatin

Membrane‐less organelles are thought to form through the nucleation of protein and nucleic acid scaffolds, which will be enriched in the phase‐separated compartment, compared with the surrounding solution 20. A key parameter determining the composition of the droplet is the scaffold's concentration 51. The scaffold proteins that mediate phase separation often contain IDRs, thought to be important for nucleating liquid droplets 29, 52, 53, 54, 55. However, IDRs can be present in “nucleating” components as well as “recruited” components. Most attention in the field has been devoted to IDRs, but it is important to keep in mind that structured domains may also contribute to phase separation.

**Figure 1:**
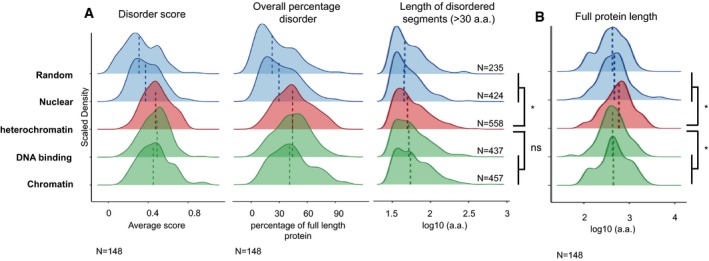
Analysis of the disorder content of the selected heterochromatin proteins Analysis of three factors to measure disorder behaviour using both the PONDR‐VLXT and IUPRED predictors. In the left panel, the disorder score per protein. In the centre panel, the percentage of predicted disorder per protein. In the right panel, the lengths of the predicted disordered regions for each protein set (length of disordered segments (> 30 a.a.)). For length of disordered regions, segments shorter than 30 amino acids were removed (based on Forma‐Kay *et al* 56 and Ward *et al* 105). The 148 heterochromatin proteins were compared with control protein sets of the same number generated from random sampling of chromatin, nuclear, DNA binding or total proteomes. The dotted lines correspond to the median value for the distributions shown. *P ≤ 0.05 and ns > 0.05 by two‐sided unpaired Wilcoxon rank‐sum test.Length in amino acids of the proteins analysed in the indicated groups. The 148 heterochromatin proteins were compared with control protein sets of the same number generated from random sampling of chromatin, nuclear, DNA binding or total proteomes. The dotted lines correspond to the median value for the distributions shown. *P ≤ 0.05 and ns > 0.05 by two‐sided unpaired Wilcoxon rank‐sum test.

IDRs are structural features of protein domains, which are often found in linker regions between folded domains as well as in post‐translational modification sites, lack a unique three‐dimensional structure and tend to have low‐complexity sequences 56, 57. IDRs are thought to drive liquid–liquid phase separation by forming multivalent interactions through their amino acid side chains 19. We asked whether the heterochromatin proteins that we identified have a higher propensity to exhibit disorder properties or IDRs. To characterise the potential of the 148 proteins to contribute to heterochromatin phase separation, we generated disorder estimates for them using two prediction algorithms, PONDR‐VLXT 58 and IUPRED 59. IUPRED and PONDR take into account the context of individual amino acids to calculate disorder scores for each amino acid in a given protein context. The predicted scores are thus presented as percentage disorder, mean disorder and length of disordered segments. The results obtained with both predictors were not always similar. However, the tendency was the same, and therefore, we averaged the results obtained with both algorithms. Heterochromatin proteins displayed a significantly higher disorder score, as compared to either a random group of total proteins or nuclear proteins of the same size (median = 0.47, compared with 0.31 and 0.37, respectively; Fig 1A). The median percentage length of disordered domains, measured as percentage of amino acids of the total protein length, was 44% (Fig 1A), which is similar to the percentages calculated for the proteome of several phase‐separated membrane‐less organelles and is higher than the value for organised structures such as the proteasome 60. In addition, the percentage of the protein (length) containing disordered domains was also significantly higher compared with a random (22%) or the nuclear (30%) set of proteins, indicating that heterochromatin proteins are more disordered than a random set of proteins or compared with nuclear proteins in general. Interestingly, not only the percentage of amino acids within disorder domains but also the length of disorder domains was significantly higher in the heterochromatin group of proteins (Fig 1A). Of note, heterochromatin proteins tend to be longer, compared with both groups of proteins, but also when compared with a set of global chromatin proteins or of DNA‐binding proteins (Fig 1B). The comparisons with the proteins constituting the nuclear protein groups clearly show that the subset of heterochromatin proteins displays features consistent with higher disorder scores.

We then asked whether this feature is exclusive to heterochromatin proteins or whether chromatin proteins in general and DNA‐binding proteins possess IDRs as well. For this, we calculated disorder scores, overall percentage (in a.a.) disorder and length of disorder segments for these two additional groups of proteins. Interestingly, our analyses revealed that proteins with the potential to bind DNA and chromatin have a higher disorder score as calculated using IUPRED and PONDR‐VLXT predictors, as well as higher overall percentage disorder score, compared with a random set of proteins, or to nuclear proteins (Fig 1A). We conclude that the specific part of the nucleome, which constitutes the chromatin and has the ability to bind DNA, has a higher potential to phase separate, based on IDR constitution.

To further assess the possible phase separation propensity of the 148 proteins, we used a different predictor for phase separation based on potential planar protein–protein contacts 61 (not shown). In fact, 38 of them were predicted to have a propensity to reversibly and dynamically self‐associate. However, this predictor only takes the planar Pi‐Pi interactions into consideration and further in‐depth analysis of other interactions is typically required in order to better predict phase separation propensity. HP1α, for example, which is known to phase separate, was not present in this list of proteins predicted to self‐associate, advocating the use of several features in parallel when making predictions for phase separation potential.

Further to IDRs, interactions between amino acids with opposing charges as well as cation–pi interactions are likely to play a role in liquid droplet formation 54. Molecular interactions between positively charged amino acids with nucleic acids also certainly play a role in the establishment of membrane‐less organelles enriched in RNA and RNA‐binding proteins 55, 62. In agreement with the importance of electrostatic interactions between macromolecules with different charges, phosphorylation and acetylation have been shown to perturb phase separation and dissolve membrane‐less organelles 62, 63, 64, 65. Hydrophobic interactions have also been suggested to play an important role in phase separation 35, 66. Pi‐Pi interactions between aromatic amino acids (Phe, Tyr, Trp and His) but also amino acids containing amide (Asn, Gln), carboxyl (Glu, Asp) or guanidinium (Arg) groups in their sidechain as well as amino acids with exposed backbone peptide bonds (Gly, Ser, Thr and Pro) are relevant for phase separation mediated by IDRs 61. Tyrosines and arginines have, for example, been shown to play a predominant role in the liquid droplet formation by the FUS family proteins 67.

We thus undertook a more thorough analysis of all these features. For this, we aimed to generate a more restricted group of “bona fide” heterochromatin proteins, whose location in chromocentres and/or impact on heterochromatin functions have been validated by cell biological or genetic experiments. Specifically, we used a set of proteins identified as enriched at major satellites by PiCH in mouse embryonic stem cells 68. From these, we selected those proteins, which are lost from the major satellites upon Suv39h1/h2 depletion, and which had been identified as suppressors of variegation (Su(var)) and modifiers of murine metastable epialleles (Mommes). This led to a list of seven proteins: CBX1 (HP1β), CBX5 (HP1α), ATRX, UHRF1, DNMT1, SUV420H2 and SUV39H2 (Table EV2). Excepting SUV420H2 and SUV39H2, the remaining five proteins exhibited disorder scores and overall percentage disorder values higher than the median values of the random set and nuclear proteomes (Table EV2).

**Figure 2:**
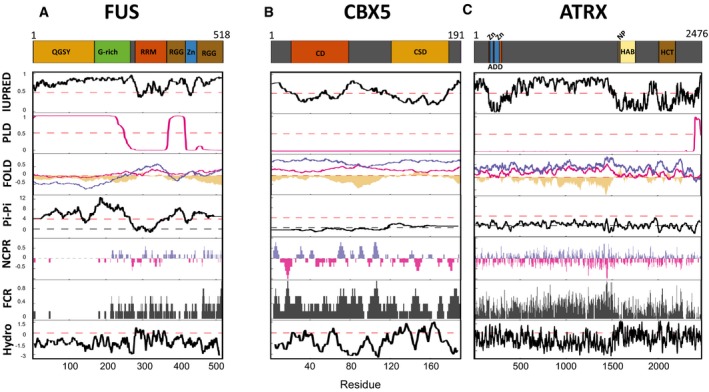
In‐depth analysis of phase separation potential for FUS, CBX5 and ATRX The analysis of regions of protein primary sequence potentially contributing to liquid–liquid phase separation for FUS, CBX5 and ATRX (see also Fig EV1) was implemented following the same methodology as published in Alberti *et al* 101. At the top, a schematic representation of the proteins is shown highlighting the different domains catalogued in UniProt. IUPRED; intrinsic disorder prediction using the IUPRED algorithm where values above 0.5 are considered disordered. PLD; prion‐like domain prediction using the PLAAC algorithm where a value above 0.5 is considered a prion‐like domain. FOLD; intrinsic disorder prediction with PLAAC (pink) or the PAPA (purple) algorithms and the fold index (yellow). Pi‐Pi; phase separation predictor based on propensity for Pi‐Pi contacts where a region of a protein is predicted to phase separate when its mean value is above 4. NCPR; net charge per residue and FCR; fraction of charged residues (sliding window of 5 using the localCIDER version 0.1.14). Hydro; hydrophobicity (sliding window of 9 using the Kyte and Doolittle scale).
For FUS, the following domains or regions are depicted: QGSY, glutamine/glycine/serine/tyrosine‐rich region (yellow); G‐rich, glycine‐rich region (green); RRM, RNA recognition motif domain (orange); RGG, arginine/glycine‐rich region (brown); Zn, zinc finger domain (blue).For CBX5, the chromo (CD in orange) and the chromo shadow (CSD in yellow) domains are shown.For ATRX, the following domains or regions are depicted: ADD, ATRX‐Dnmt3‐Dnmt3L domain (orange); Zn, zinc finger domains (blue); HAB, helicase ATP binding (beige); NP: nucleotide (ATP) binding (red); HCT, helicase C‐terminal (brown).

**Figure EV1:**
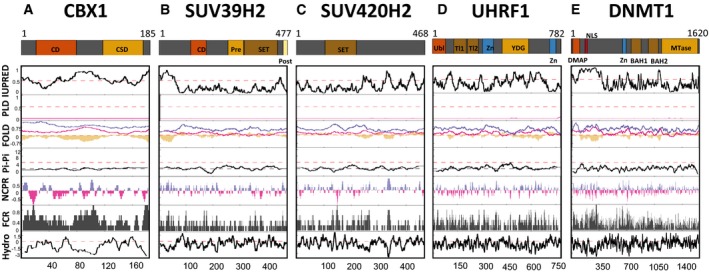
In‐depth analysis of phase separation potential for the bona fide heterochromatin proteins The analysis of regions of protein primary sequence potentially contributing to liquid–liquid phase separation for CBX1, SUV39H2, SUV420H2, UHRF1 and DNMT1 was implemented following the same methodology as in Fig 2.
For CBX1, the chromo (CD in orange) and the chromo shadow (CSD in yellow) domains are shown.For SUV39H2, the following domains or regions are depicted: CD, chromodomain (orange); Pre, Pre‐SET domain (yellow); SET, SET domain (brown); Post, Post‐SET domain (beige).For SUV420H2, the SET domain (brown).For UHRF1, the following domains or regions are depicted: Ubl, ubiquitin‐like domain (orange); Tl1 and Tl2, Tudor‐like 1 and 2 regions (brown); Zn, zinc finger domains (blue); YDG, YDG domain (yellow).For DNMT1, the following domains or regions are depicted: DMAP, DMAP‐interaction domain (orange); NLS, nuclear localisation signal (red); Zn, zinc finger domain (blue); BAH1 and BAH2, bromo‐adjacent homology 1 and 2 domains (brown); Mtase, SAM‐dependent Mtase C5 type (yellow).

We then expanded our analysis to other features indicative of a potential to phase separate, including IUPRED and FOLD disorder scores, presence of predicted prion‐like domains, propensity for Pi‐Pi contacts, fraction of charged residues and net charge per residues across each protein as well as hydrophobicity (Figs 2A–C and EV1A–E). In addition, to provide a relevant comparison, we performed the same analysis with the transcription factor FUS (Fig 2A), which has been shown to phase separate both *in vitro* and *in vivo* 67, 69. This uncovered, for example, a clear prion‐like domain (PLD) in ATRX as well as high IUPRED scores in ATRX, but also in CBX5 (Fig 2B and C), as previously reported 17. Additionally, the N‐terminal domain of SUV39H2, known to interact with RNA, exhibited also high IUPRED score (Fig EV1B). Interestingly, SUV39H2 is highly enriched in mouse zygotes 70, and therefore, the study of its role in heterochromatin formation, and potentially in phase separation, *in vivo*, should be an exciting research avenue. We find that the “bona fide” heterochromatin proteins contain various segments of high hydrophobicity and with a high fraction of charged residues (Figs 2A–C and EV1A–E), which could potentially favour phase separation. These features may be hard to interpret however, since they may not be sufficient *per se* to drive liquid–liquid phase separation, as recently shown for the FUS low‐complexity domain 69. Overall, these analyses suggest that the “bona fide” heterochromatin proteins that we selected have additional features linked to the potential to phase separate.

The above biophysical and biochemical characteristics are in general used as a proxy to assess if a given molecular—and in some instances cellular—process could be explained by phase separation. However, they are only an indicator. In fact, local concentration and post‐translational modifications are key. For example, in HP1α, phosphorylation is required for structural changes that promote phase separation 16. While such additional features should be taken into account, overall, our analysis reveals that several proteins associated biochemically with constitutive heterochromatin present characteristics of proteins within membrane‐less organelles and some of them are predicted to phase separate.

### Establishment of heterochromatin *in vivo*

A significant rearrangement and reprogramming of constitutive heterochromatin occurs during germ cell and subsequently early embryonic development 71, 72. During preimplantation development, H3K9me3 is dramatically decreased and re‐established on both parental genomes, albeit with different temporal dynamics 73, 74, 75, while H4K20me3 and H3K64me3, two modifications downstream of H3K9me3 76, are both removed at the 2‐cell stage and not re‐established until post‐implantation 11, 77. In addition, chromocentres only emerge from the late 2‐cell stage onwards, while HP1α, the primary heterochromatin protein suggested to be responsible for its phase separation 16, 17, is not thought to be expressed during preimplantation development 78.

We suggest that in order to understand the role of phase separation in heterochromatin function, it will be particularly revealing to describe the dynamics of phase‐separated heterochromatin during these periods of development, when heterochromatin is dynamic. In addition, a clearer temporal correlation could be made between the known markers of heterochromatin and the phase‐separated heterochromatin state. For example, which, if any, histone modifications or protein readers typical of classical constitutive heterochromatin (such as H3K9me3, H4K20me3 and HP1 isoforms) or features such as chromocentres, temporally and spatially correlate with the appearance of a phase‐separated heterochromatic state?

**Figure 3:**
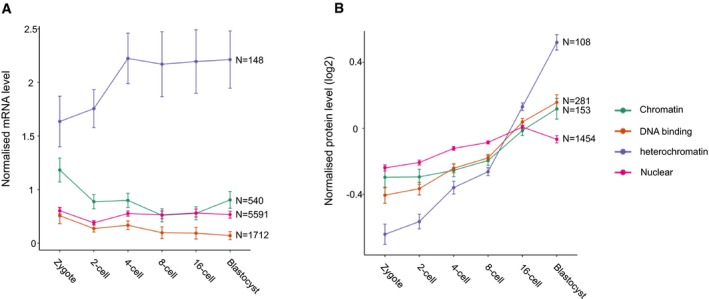
mRNA and protein levels of the selected heterochromatin and control datasets during mouse preimplantation embryonic development Mean ± SEM mRNA levels normalised to the sum of expression across detected genes during preimplantation development (data from Deng *et al*). The analysis was carried out for the 148 genes coding for the heterochromatin proteins as well as all the genes with “chromatin” (540), “DNA binding” (1,712) or “nuclear” (5,591) in their GO terms.Mean ± SEM protein levels during preimplantation development by mass spectrometry, normalised to average expression of all detected proteins (data from Gao *et al*). The analysis was carried out for the 108 detected heterochromatin proteins as well as all the proteins with “chromatin” (153), “DNA binding” (281) or “nuclear” (1,454) in the GO terms of their corresponding genes.

Can we predict phase transition occurrence during mouse preimplantation development? We reasoned that an analysis of the patterns of expression of heterochromatin proteins that we identified (Table EV1) during these stages of development, in combination with the knowledge of their predicted phase separation properties, can give a first forecast of the dynamics of phase‐separated heterochromatin in mouse embryos. An analysis of publicly available RNAseq datasets 79 indicated a clear average upregulation of the genes encoding the 148 heterochromatin proteins at the 4‐cell stage (Fig 3A). This suggests firstly that, for the most part, these genes do not exhibit the typical dynamics of maternally inherited transcripts, a fact not insignificant considering the large pool of such transcripts. Additionally, this trend was markedly different to the expression dynamics of the other groups of genes analysed, which included genes coding for chromatin proteins, in general, DNA‐binding proteins, as well as the complete nucleome (Fig 3A). Thus, it is likely that constitutive heterochromatin is largely remodelled after fertilisation, fitting with the known dynamics of heterochromatin markers by immunostaining and of H3K9me3 ChIPseq 74, 80. Interestingly, the timing of this increase also correlates with the reported increase in chromatin compaction between the 2‐cell and 8‐cell stages 81, 82 and the establishment of chromocentres from the late 2‐cell stage 83.

Analysis of mass spectrometry data 84 showed that the 108 (73%) of 148 heterochromatin proteins detected displayed a collective increase in protein levels towards the blastocyst stage (Fig 3B). In fact, this tendency is more consistent at the protein level than for the mRNA levels. The heterochromatin proteins displayed increasing expression over the preimplantation period, with a clear, sharp increase after the 8‐cell stage (Fig 3B). Thus, constitutive heterochromatin may gradually mature during the early period of mammalian development. While we did not observe any correlation between the degree of predicted disorder and expression level (not shown), the clear increase in both mRNA (at the 4‐cell stage) and protein (at the morula–blastocyst stage) suggests that the proteins more likely to promote heterochromatic phase separation are on average expressed at later timepoints during mouse preimplantation development. Thus, heterochromatin, which is atypical in numerous other aspects in the period of development immediately after fertilisation 85, may also not phase separate at this stage. Potentially, phase separation of heterochromatin only occurs as it matures, after chromocentre formation at the late 2‐cell stage, and chromatin compaction and silencing of repetitive elements at the 8‐cell stage. It will be interesting to determine the point at which heterochromatin is able to initiate phase separation and its functional contribution to the embryo.

### Current *in vivo* assays to address phase separation in heterochromatin establishment

To date, all methods to study phase separation *in vivo* are microscopy‐based, primarily using differential interference contrast microscopy or fluorescence microscopy to visualise the sphericity, number and dynamics of condensates 20, 21, 54. Indeed, the liquid state of a membrane‐less organelle can be called by demonstrating their ability to fuse or fission 17, 21, 27, 28, 33, 64. Fluorescence recovery after photobleaching (FRAP) can also be used to determine whether proteins diffuse inside the phase‐separated compartment as well as between the surrounding environment 18. Some studies target part of the membrane‐less organelle in order to assess internal diffusion of tagged proteins of interest 20, 27. In addition, bleaching the whole condensate assesses the diffusion of the protein of interest between the condensate and its environment 28, 33, 36, 63, 86. Importantly, FRAP has been used to measure the mobile and immobile HP1α fractions to uncover liquid‐like properties of heterochromatin in the developing *Drosophila* embryo 17.

Imaging analyses are in general amenable to early mouse embryos, but phototoxicity is a major problem and must be taken into consideration when used in live embryos. FRAP has previously been used to study dynamics of histone proteins during preimplantation development 81, 87. Therefore, implementation of FRAP and differential interference contrast microscopy in embryos could address whether specific proteins and/or compartments can fuse, as well as determine diffusion dynamics, which has been done for, e.g., transcription factors 88. However, additional strategies requiring higher photon absorption, such as the number and brightness (N&B) 89 and raster image correlation spectroscopy (RICS) 90, will require major adaptation. Indeed, the N&B method was used in *Drosophila* to show that HP1α exhibits coordinated movement at the heterochromatin boundary, while the RICS method showed that HP1α diffusion was slower in heterochromatin. As both of these observations are predicted to occur at the boundary of a liquid condensate, it was concluded that HP1α dynamics are consistent with the heterochromatin domains being in a liquid state 17.

The liquid state of condensates *in vivo* can also be assessed using 1,6‐hexanediol, an aliphatic alcohol that disturbs weak hydrophobic interactions and thus liquid condensates 91. However, this compound can be rather toxic for eukaryotic cells and is therefore typically used within short time windows 17, 33. Mutating amino acids necessary for phase separation of the protein of interest may be another strategy to manipulate liquid condensates *in vivo*, in order to probe function. This has been done, for example, by mutating the tyrosines to serines in the IDR of FUS, which disturbs phase separation of FUS 64, 92. Modifying relevant serines and threonines to glutamic acid, which mimics phosphorylation, is also another means of the disturbing phase separation 64, 93. Acetylation of intrinsically disordered regions has also been shown to regulate phase separation 65 and mimicking acetylation may provide additional experimental strategies.

Finally, it is important to note that we have not considered a possible role for RNA interactions in this current work. Membrane‐less organelles are enriched in RNAs and RNA‐binding proteins 60, 94. The role of RNA interactions in phase separation has been extensively characterised *in vitro*, as well as *in silico*, and less often *in vivo*. Ribosomal RNA transcription, for example, regulates nucleoli assembly 95. In *C. elegans,* P granule formation has been suggested to be mediated by interactions between mRNA and the PGL‐3 protein 96. mRNA also controls the phase behaviour of RNA‐binding proteins such as TDP43 and FUS, which will form liquid droplets or solid aggregates depending on mRNA availability 97. Several RNA‐binding proteins have the ability to phase separate, such as the heterogeneous nuclear ribonucleoproteins hnRNPA1 and hnRNPA2 28, 98. In this context, it is important to note that major satellites are robustly transcribed in zygotes and 2‐cell stage embryos 80, 99, 100. This raises the interesting possibility that this RNA may be a good candidate as a scaffold for phase‐separated domains *in vivo* in the mouse embryo.

The plethora of these studies, as well as the nature of the open questions to address how, when and under which conditions heterochromatin phase separates *in vivo*, promises exciting research in the years to come. From the technical viewpoint, it will be important to define the standards of the experimental approaches used to study phase separation *in vivo*, as recently proposed 101. From the developmental perspective, it will be exciting to apply different methodologies to determine whether and when phase separation regulates establishment of heterochromatin.

## Materials and Methods

### Merging mass spectrometry datasets

Unless otherwise stated, all analyses were performed in R studio (version 1.2.1335) with the R version (R version 3.5.2 (2018‐12‐20)). The bioinformatic analysis was based on 7 mass spectrometry studies performed in mammalian cells 6, 39, 40, 41, 42, 43, 44. Proteins predicted to be heterochromatic were selected based on their ability to bind H3K9me3, H3K9me3‐modified nucleosomes with and without DNA methylation, or to their enrichment in the sonication resistance fragment of the chromatin. Due to the little overlap between the mass spectrometry studies, the proteins present in more than one mass spectrometry study were kept for the analysis. Note that the antibodies used in these studies have been thoroughly characterised, as follows: Bartke, Becker, Engelen, Ji and Soldi all used the same antibody (Abcam ab8898), which was reported to be highly specific to H3K9me3, with no binding to H3K9me2 or H3K9me1, with only a slight cross‐reactivity to H3K27me3. The two other studies used H3K9me3 peptides as bait in pulldowns.

### Identification of orthologs across model organisms

The orthologs in *D. rerio*,* D. melanogaster* and *C. elegans* were identified using the Ensembl project website with the Ensembl release 94 102 and downloading a dataset with the orthologs in the different species of the mouse genes (GRCm38.p6). For the *S. pombe* orthologs, a dataset containing the human orthologs of *S. pombe* orthologs was downloaded from the PomBase project website 103.

### Disorder analysis

The control groups for the analysis of disorder content were selected by retrieving, from the Ensembl project website with the Ensembl release 96 102, all the mouse genes (GRCm38.p6) or the ones which have chromatin, nuclear or DNA binding in their GO Term Names. All the genes also present in the heterochromatin dataset were later removed from these control groups. In order to compare the control and the heterochromatin groups, 148 genes were randomly sampled without replacement from each of the control datasets to obtain the final control groups. The fasta files from all the selected proteins were then downloaded from NCBI using the efetch function of the Entrez package build in Biopython 104. To calculate the length in amino acids of the proteins in each group, the fasta files were imported in Rstudio with the read.fasta function of the seqinr package (version 3.4.5). For the disorder analysis, disorder estimates were generated for the proteins in the different groups using two prediction algorithms, PONDR‐VLXT 58 and IUPred2 long disorder 59. The predictors give a value between 0 and 1 for each amino acid where above 0.5 is predicted to lie within a disordered region. For each predictor, the average value (average disorder score) and the percentage of amino acids with a value over 0.5 (overall percentage disorder) were then calculated for each protein. The latter analysis was done on the average of the values obtained with the two predictors. The analysis of the length of the disorder fragments was done with the PONDR‐VLXT. This was done by counting the number of predicted disorder fragments of different size in amino acids across the different proteins of the same group. For length of disordered regions, segments shorter than 30 amino acids were removed (based on Forman‐kay *et al* and Ward *et al* 56, 105). To assess the statistical significance of the difference between the heterochromatic group and the different control groups, a two‐sided unpaired Wilcoxon rank‐sum test was performed in R with the wilcox.test function with default settings, as the data were found to be non‐parametric. All the plots were done using ggplot2.

### Analysis of bona fide heterochromatin proteins

The 7 bona fide heterochromatin proteins were selected based on their specific association to major satellite genomic regions as described by Saksouk *et al* 68. Briefly, proteins enriched at major satellite genomic regions, and therefore constitutive heterochromatin, were identified by proteomics of isolated chromatin segments (PiCH) in mouse embryonic stem cells. The 7 bona fide heterochromatin proteins are depleted at major satellites when Suv39h1 and Suv39h2 are knocked out and have been identified as suppressors of variegation and modifiers of murine metastable epialleles.

The drawProteins (version 1.2.0) package was used to obtain the features of the 7 bona fide heterochromatin proteins from the UniProt Features API. The prediction of intrinsic disorder was done with the IUPred2 long‐disorder algorithm 59. The prion‐like domains were predicted with the PLAAC algorithm using the website (http://plaac.wi.mit.edu) with the default settings 106. The intrinsic disorder prediction with the PLAAC, the PAPA and the fold index was obtained with the same website. To predict the phase separation property of the 7 bona fide heterochromatin proteins based on propensity for Pi‐Pi contacts, the Pi‐Pi predictor was used online on the Forman‐Kay's laboratory website 61. The net charge per residue and the fraction of charged residues were obtained using the localCIDER (version 0.1.14) 107 with a sliding window of 5. The hydrophobicity was calculated with the ExPASy website 108 with the Kyte and Doolittle scale 109 and a sliding window of 9. All the plots shown in Figs 2 and EV1 were done with ggplot2.

### Analysis of gene expression in mouse preimplantation embryos

RNAseq dataset previously published 79 was analysed downloading the expression matrix provided in a GitHub repository (“jhsiao999/singleCellRNASeqMouseDengESC”) which contains the data from National Center for Biotechnology Information Gene Expression Omnibus (“GSE45719”). The expression matrix was later normalised by library size by dividing the counts by the sum of expression across detected genes in each sample. Heterochromatin (148), chromatin (540), DNA binding (1,712) or nuclear (5,591) genes were extracted from the datasets based on GO terms, excepting for the “heterochromatin” dataset, which was selected in the current study as described above. The mean normalised mRNA levels and standard errors for each gene group and embryonic development stage were plotted using ggplot2.

### Analysis of protein levels in mouse preimplantation embryos

The mass spectrometry study of preimplantation development by Gao *et al* 84 was analysed to investigate the expression pattern of the heterochromatin (106) and control groups. The control groups correspond to all the proteins with chromatin (153), DNA binding (281) or nuclear (1,454) in the GO terms of their corresponding genes. The protein levels were normalised to average expression of all detected proteins in each sample and transformed to a base 2 logarithmic scale. The normalised mean protein expression levels and standard errors for each protein group and embryonic development stage were plotted using ggplot2.

## Author contributions

MG, AB and M‐ET‐P conceived the work and wrote the manuscript. MG performed bioinformatic analyses. AB and M‐ET‐P supervised the work.

## Conflict of interest

The authors declare that they have no conflict of interest.

## Supporting information



Expanded View Figures PDF

Table EV1

Table EV2

Review Process File
