# Supplementary material for: Expression and phase separation potential of heterochromatin proteins during early mouse development
Source: EMBO Rep. 2019 Nov 7;20(12):e47952. doi: 10.15252/embr.201947952 (PMC6893284; doi:10.15252/embr.201947952)
Supplement: Supplementary file 1 — Expanded View Figures PDF [file EMBR-20-e47952-s001.pdf]

Expanded View Figures

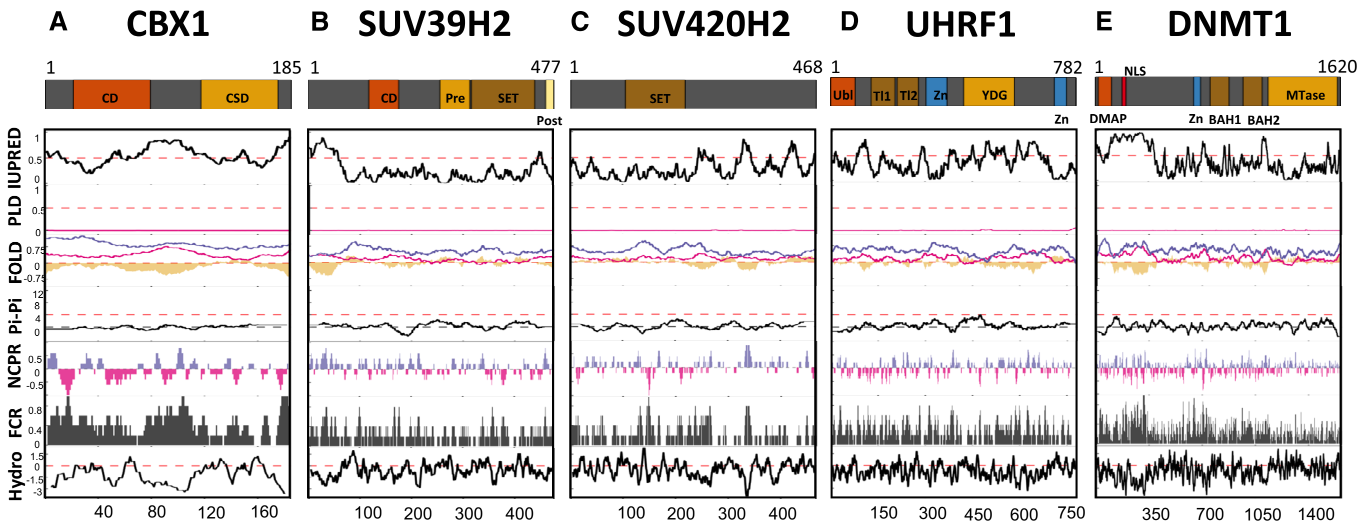

Figure EV1. In-depth analysis of phase separation potential for the bona fide heterochromatin proteins.

The analysis of regions of protein primary sequence potentially contributing to liquid–liquid phase separation for CBX1, SUV39H2, SUV420H2, UHRF1 and DNMT1 was implemented following the same methodology as in Fig 2.

A For CBX1, the chromo (CD in orange) and the chromo shadow (CSD in yellow) domains are shown.

B For SUV39H2, the following domains or regions are depicted: CD, chromodomain (orange); Pre, Pre-SET domain (yellow); SET, SET domain (brown); Post, Post-SET domain (beige).

C For SUV420H2, the SET domain (brown).

D For UHRF1, the following domains or regions are depicted: Ubl, ubiquitin-like domain (orange); Tl1 and Tl2, Tudor-like 1 and 2 regions (brown); Zn, zinc finger domains (blue); YDG, YDG domain (yellow).

E For DNMT1, the following domains or regions are depicted: DMAP, DMAP-interaction domain (orange); NLS, nuclear localisation signal (red); Zn, zinc finger domain (blue); BAH1 and BAH2, bromo-adjacent homology 1 and 2 domains (brown); Mtase, SAM-dependent Mtase C5 type (yellow).

© 2019 The Authors
