# Supplementary material for: Expression and phase separation potential of heterochromatin proteins during early mouse development
Source: EMBO Rep. 2019 Nov 7;20(12):e47952. doi: 10.15252/embr.201947952 (PMC6893284; doi:10.15252/embr.201947952)
Supplement: Supplementary file 2 — Table EV1 [file EMBR-20-e47952-s002.docx]

**Table EV1. Conserved heterochromatin components.**

List of the 148 heterochromatic proteins identified in more than one mass spectrometry study in mammalian cells [6,39-44] (Gene information). The number of studies in which the protein was found is shown under the Mammals column. The number of orthologs found in *D. renio*, *S.pombe*, *D. melanogaster* and *C. elegans* can be seen in the Orthologs column. This analysis was done using the Ensembl project website with the Ensembl release 94 [102] for the orthologs in *D. renio*, *D. melanogaster* and *C. elegans,* and with the PomBase project website [103] for *S.pombe*. The two Disorder estimates show the disorder score and overall percentage disorder obtained with the PONDR-VLXT and IUPRED predictors.
