# Supplementary material for: Expression and phase separation potential of heterochromatin proteins during early mouse development
Source: EMBO Rep. 2019 Nov 7;20(12):e47952. doi: 10.15252/embr.201947952 (PMC6893284; doi:10.15252/embr.201947952)
Supplement: Supplementary file 3 — Table EV2 [file EMBR-20-e47952-s003.docx]

|  | **Protein length** | **disorder score** | **Overall percentage disorder** |
| --- | --- | --- | --- |
| **Heterochromatin**  **(median)** | 615.5 | 0.47 | 44.32 |
| ***CBX5*** | *191* | *0.5* | *45.55* |
| ***CBX1*** | *185* | *0.54* | *54.86* |
| ***ATRX*** | *2476* | *0.56* | *58.70* |
| **DNMT1** | 1620 | 0.39 | 33.36 |
| **UHRF1** | 782 | 0.39 | 34.91 |
| **SUV39H2** | 477 | 0.28 | 20.86 |
| **SUV420H2** | 468 | 0.32 | 22.86 |

**Table EV2: Disorder analysis of bona fide heterochromatic proteins**

Disorder analysis for the 148 heterochromatin proteins (median) and the 7 ‘bona fide’ heterochromatic proteins. The first column shows the protein length in amino acids. The second and third column show the disorder score and overall percentage disorder calculated using the PONDR-VLXT and IUPRED predictors similarly to Figure 1.
