# Supplementary material for: Expression and phase separation potential of heterochromatin proteins during early mouse development
Source: EMBO Rep. 2019 Nov 7;20(12):e47952. doi: 10.15252/embr.201947952 (PMC6893284; doi:10.15252/embr.201947952)
Supplement: Supplementary file 4 — Review Process File [file EMBR-20-e47952-s004.pdf]

EMBO
reports

Expression and phase separation potential of heterochromatin proteins during early mouse development

Manuel Guthmann, Adam Burton & Maria-Elena Torres-Padilla

Review timeline:

Submission date:	19th Feb 2019
Editorial Decision:	24th May 2019
Revision received:	30th Aug 2019
Editorial Decision:	30th Sep 2019
Revision received:	3rd Oct 2019
Accepted:	16th Oct 2019

Editor: Esther Schnapp

Transaction Report:

(Note: No Peer Review Process File is available with this article, as the authors have chosen not to make the review process public in this case.)
